# The role of parenting stress in anxiety and sleep outcomes in toddlers with congenital heart disease

**DOI:** 10.3389/fped.2022.1055526

**Published:** 2023-01-06

**Authors:** Charles Lepage, Isabelle Gaudet, Amélie Doussau, Marie-Claude Vinay, Charlotte Gagner, Zorina von Siebenthal, Nancy Poirier, Marie-Noëlle Simard, Natacha Paquette, Anne Gallagher

**Affiliations:** ^1^ Research Center, Sainte-Justine University Hospital Research Center, Montréal, QC, Canada; ^2^ Department of Psychology, Université de Montréal, Montréal, QC, Canada; ^3^ Clinique d’investigation neurocardiaque (CINC), Sainte-Justine University Hospital, Montréal, QC, Canada; ^4^ Department of Surgery, Division of Cardiac Surgery, Faculty of Medicine, Université de Montréal, Montréal, QC, Canada; ^5^ School of Rehabilitation, Faculty of Medicine, Université de Montréal, Montréal, QC, Canada

**Keywords:** CHD (congenital heart disease), neurodevelopement, quality of sleep, parenting stress, behavior, anxiety, early assessment, psychosocial care

## Abstract

**Objectives:**

This retrospective cohort study investigates how parenting stress, measured at 4 months of age by use of a classic three-dimensional parent-reported scale (Parenting Stress Index, 4th Ed. or PSI-4), can predict anxiety symptoms and quality of sleep at 24 months in toddlers with congenital heart disease (CHD).

**Study Design:**

Sixty-six toddlers with CHD followed at our cardiac neurodevelopmental follow-up clinic were included in this study. As part of their systematic developmental assessment program, parents completed questionnaires on their stress level (PSI-4) when their child was 4 months old, and on their child's anxiety symptoms and quality of sleep at 24 months. Eight multiple linear regression models were built on the two measures collected at 24 months using the PSI-4 scores collected at 4 months. For each measure, four models were built from the PSI-4 total score and its three subscales (Parental Distress, Parent-Child Dysfunctional Interaction, Difficult Child), controlling for sex and socioeconomic status.

**Results:**

The PSI-4 Difficult Child subscale, which focuses on parenting anxiety related to the child's behavioral problems and poor psychosocial adjustment, accounted for 17% of the child's anxiety symptoms at 24 months. The two other PSI-4 subscales (Parental Distress and Parent-Child Dysfunctional Interaction) and the PSI-4 total score did not contribute significantly to the models. None of the four regression models on perceived quality of sleep were significant. It is important to note that 33% of parents responded defensively to the PSI-4.

**Conclusions:**

Parenting stress related to the child's behavioral problems and poor psychosocial adjustment, measured when the child is 4 months old, is associated with the child's ulterior anxiety symptoms. As very few standardized tools are available to assess the behavioral and psychoaffective development of infants, this study highlights the importance of early psychosocial screening in parents of infants with CHD. The high rate of significant Defensive Responding Indices reminds us to not take parent reports at face value, as their actual stress levels might be higher.

## 1. Introduction

Children with congenital heart disease (CHD) are at high risk for neurodevelopmental impairments, including behavioral and psychoaffective deficits, which can alter their developmental trajectory and quality of life (1). More specifically, school-age children with CHD seem to display more anxiety and depressive symptoms than their healthy peers (2). Gupta and al (3). found that covert anxiety, fear, depression and behavioral problems were more prevalent in children with CHD who were not exhibiting clinically significant psychosocial impairments, compared to the normative population. They also found that school-age children with cyanotic CHD displayed more physiological signs of anxiety (i.e., shortness of breath, increased heart rate, sweaty hands) than healthy children, which was associated with isolation and lower self-esteem, thus greatly impacting those children's relationships with their family and same-age peers. Even preschool-age children with CHD have 5–7 times higher odds of developing an anxiety disorder (4). This highlights the relevance of examining anxiety outcomes in young children with CHD.

Meanwhile, up to 50% of parents of children with CHD exhibit significant emotional distress (5). During the perinatal period, parents of children with CHD experience considerable stress due to their child's life-threatening medical condition, multiple surgeries, long hospitalizations, and neurodevelopmental and behavioral challenges (6, 7), thus making it harder for them to thrive in their parental role (8, 9). It has also been shown that parents may pass on their anxiety to their child, through overprotective behavior, fears and personal or interpersonal distress (10). Therefore, the more anxious the parent, the more behavioral and psychoaffective problems they tend to identify in their child, and the higher the chances that their child will develop a similar anxious profile (3). Considering the high risk for emotional distress in parents of children with CHD, the relationship between parenting stress and the child's anxiety ought to be further investigated.

Visconti et al. (11) conducted one of the first longitudinal studies on the effects of parenting stress on behavioral and psychoaffective adjustment in children with dextro-transposition of the great arteries (d-TGA) and they found that parents with more stress at 12 months reported more behavioral problems in their children 3 years later. Also using a longitudinal design, Hsiao et al. (12) identified five distinct evolutive patterns of behavioral and psychoaffective difficulties in children with CHD over a two-year period (participants enrolled between the ages of 1,5–10 years old): “persistent normal,” “initial problematic,” “worsening,” “persistent problematic,” and “subclinical.” Children of all ages whose parents had higher levels of parenting stress were more likely to be categorized into the “initial problematic,” “subclinical,” and “persistent problematic” patterns, suggesting that behavioral and psychoaffective functioning is influenced by early factors including parenting stress. Altogether, these studies highlight the importance of early detection of parenting stress, in order to promote healthy behavioral and psychoaffective development in children with all types of CHD (13).

High levels of parenting stress have also been associated with more sleep problems in healthy 5-year-olds who exhibit behavioral and psychoaffective problems (14), as well as in ill children (15). It has also been documented that children with CHD are at high-risk of developing sleep pathologies (16) or disrupted sleep patterns, especially when there is increased medical complexity, longer hospitalization stays and lower parental education (17). Sleep plays a fundamental role in the child's development (18, 19), and it has been reported to be among the parent's main concerns (20) due to the adverse effects of sleep deprivation on the child's daily functioning. Since parents of children with CHD experience very high levels of stress (6, 7), it is relevant to examine the relationship between parenting stress and the child's quality of sleep. A better understanding of the potential implications of parenting stress would affirm the need for early preventive screening of distressed families.

Many sociodemographic and clinical factors related to CHD are known to influence neurodevelopmental outcomes (21, 22). Investigating the role of parenting stress on anxiety and sleep outcomes is essential in order to better understand the underlying contributing parental factors (23, 24), which might be modifiable. Therefore, this retrospective study aims to investigate how early parenting stress, measured at 4 months, can explain the child's anxiety symptoms and quality of sleep, measured at 24 months, in a cohort of toddlers with CHD. We expected parenting stress at 4 months to account for a significant part of the variance in anxiety and sleep outcomes at 24 months.

## 2. Materials and methods

### 2.1. Procedure and participants

We performed a retrospective examination of all patients aged 24 months and older (*n* = 281) seen at our cardiac neurodevelopmental follow-up clinic and whose parents had consented to their child's medical information being used for research purposes. In order to be included in the study, children had to have an ante- or perinatal diagnosis of CHD, have undergone at least one corrective surgery early in life (palliative or temporary procedures such as the Rashkind procedure or the installation of cardiac stents were not considered) and have in their medical chart all parent-reported questionnaires of interest completed during the systematic follow-up assessments at 4 and 24 months. More specifically, parents filled the Parenting Stress Index, 4th Edition—Short Form (PSI-4-SF) at 4 months, and the Child Behavior Checklist 1.5–5 years old (CBCL 1.5–5 years old) and the *Échelle de dépistage des troubles de sommeil pédiatriques* (HIBOU; Pediatric Sleep Disorders Screening Scale) at 24 months (see below for detailed descriptions of the parental questionnaires).

### 2.2. Variables

#### 2.2.1. Parenting stress

Parenting stress levels were measured using the PSI-4-SF (25) when patients with CHD were 4 months old. This questionnaire was completed either by one or both of the patients' parents. If both parents completed the questionnaire, the scores of one of the parents were chosen randomly during data collection (see Supplementary Table S1). The PSI-4-SF includes three subscales of 12 items each: Parental Distress (PD; example of item: “I don’t enjoy things as I used to.”), Parent-Child Dysfunctional Interaction (PCDI; example of item: “My child rarely does things for me that make me feel good.”) and Difficult Child (DC; example of item: “My child is very emotional and gets upset easily.”). The parent answers using a Likert scale with five anchors ranging from “Strongly Disagree” to “Strongly Agree.” The total stress score is calculated by combining the total scores of the three subscales. A raw score of 110 and over (85th percentile) points to a level of parenting stress that is higher than normal. The questionnaire also evaluates the parent's propensity to answer defensively (Defensive Responding Index), whether because the parent who filled out the questionnaire underestimated his or her stress level (deliberately or not) or due to an abnormally low level of parenting stress, possibly related to parental disengagement. Defensive responses are abnormally low scores (“Strongly Disagree”) on items to which most parents, not necessarily in clinical contexts, would answer that they somewhat agree. The scores of seven items of the PD subscale are combined (example of an item: “I feel trapped by my responsibilities as a parent.”) and the Defensive Responding Index is considered significant if the sum is 10 or less, meaning that the results must be interpreted with caution. The PSI-4-SF has very good psychometric properties [Cronbach's *α* superior to .80; see reference (26)] and its validity and reliability have been confirmed in various populations (27, 28).

#### 2.2.2. Anxiety

The behavioral, psychosocial and psychoaffective development of the 24-month-old patient was evaluated using the French version of the parent form of the CBCL 1.5–5 years old [*CBCL 1.5–5 ans*; see references (29, 30)]. This questionnaire includes 99 items divided in 15 subscales (i.e., “Attention Problems”, “Somatic Complaints”, “Internalizing Problems”, etc.). The raw score calculated from each subscale's items (“0 = not true; 1 = somewhat or sometimes true; 2 = very true or often true”) is converted to a T score and a percentile rank. For each subscale, an above normal or clinical cut-off score is specified. In this study, we considered the “Anxiety Problems” subscale of the questionnaire. This subscale contains 10 items and therefore has a maximum raw score of 20. A raw score of 8 and above indicates clinically significant anxiety symptoms. Validity of the CBCL 1.5–5 years old for assessing the behavioral, psychosocial and psychoaffective development of preschoolers has been corroborated using diverse populations (31, 32). Many authors have confirmed the very good psychometric properties of the questionnaire (Cronbach's *α* superior to .72 for all subscales)—see among others Ha et al. (33) and Ivanova et al. (34).

#### 2.2.3. Quality of sleep

The patient's quality of sleep at 24 months was assessed using the HIBOU (Pediatric Sleep Disorders Screening Scale). This is a non-standardized parent-reported screening tool translated into French by Godbout and Martello (35) from the original English version (36). This questionnaire has proven to be useful for clinicians, allowing them to easily collect information on the child's sleep habits, thus improving their ability to identify early on potential sleep problems and refer the child to a specialist, if needed (37). The questionnaire includes five subscales: “Irregular schedule and excessive diurnal sleepiness”, “Insomnia”, “Moves during sleep”, “Obstruction” and “Ultra-vigilance”. Each subscale refers to 1 or 2 items for which the parent provides a score ranging from 0 to 3. The total raw score ranges from 0 to 27. A total score of 16 and over indicates that the child has significant sleep difficulties and is at high risk of developing a sleep disorder, and a referral to a specialist is recommended. A total score between 10 and 15 indicates sleep difficulties that should be monitored, but that do not yet justify a referral.

### 2.3. Statistical analyses

Analyses were conducted using the Statistical Package for Social Sciences (SPSS, version 27). First, the data was explored to ensure assumptions of normality were met and Descriptive comparison statistics between participants and non-participants were performed using independent t-tests. Then, to determine the interrelatedness between parenting stress at 4 months (PSI-4 total score and PSI-4 subscale scores PD, Parental Distress; PCDI, Parent-Child Dysfunctional Interaction; and DC, Difficult Child) and our behavioral and psychoaffective measures of interest at 24 months (CBCL Anxiety Problems; HIBOU total score) bivariate Pearson correlations were computed prior to the regression models. The correlation coefficients yielded the maximum degree of linear relationship that could be obtained between our variables. Finally, multiple linear regression analyses were performed to examine how parenting stress at 4 months could explain ulterior anxiety symptoms and quality of sleep at 24 months.

Four regression models were built on the CBCL Anxiety Problems score at 24 months. The first model included the PSI-4 total stress score at 4 months as a predictor. The three other models each included a PSI-4 subscale score other than the total stress score (PD, PCDI, DC). Since significant differences were found between the PSI-4 scores of parents who responded defensively (see below) to the questionnaire and those who did not, Defensive Responding was included as a dichotomic predictor in all models. Finally, sex (38, 39), socioeconomic status (40, 41) and duration of hospitalization at first surgery (42) were controlled, as all these factors have been shown to have a significant influence on the neurodevelopment of children with CHD.

Four other identical regression models were built on the HIBOU score at 24 months. The exact same variables were controlled. Since the HIBOU questionnaire only generates a raw score, the raw scores of all three questionnaires were used. Moreover, even though the patients were between 2 and 5 years old when the analyses were conducted (*M* = 43.16 months; SD = 7.07; range of 28–58 months), they were all roughly the same age when the questionnaires were completed (*M* = 24.24 months; SD = 0.93; range of 23–29 months; see Supplementary Table S1) and were therefore part of the same age-based normative group, meaning that the raw scores represented the same level of challenges encountered. Finally, a Benjamini-Hochberg correction for multiple analyses was applied, because four comparisons were performed on each dependent variable.

## 3. Results

### 3.1. Descriptive statistics

Among the 281 patients whose medical charts were reviewed, ten were excluded because they did not require corrective surgery and 205 were excluded because of incomplete questionnaires, interruptions in the follow-up process or late referrals to the clinic (past the 4-month assessment). A total of 66 patients with CHD (42 boys; mean age 43.17 months ±7.07) were ultimately included in this study. See Supplementary Table S1 for detailed statistics of the patients' characteristics. In order to investigate if our sample was representative of the patients at our cardiac neurodevelopmental follow-up clinic, we compared socio-demographic and clinical variables between the included patients (*n* = 66) and the patients followed at the neurocardiac clinic who were excluded (*n* = 205). These results are presented in Table 1.

**Table 1:** Descriptive statistics of included and excluded patients.

Socio-demographic or medical characteristics	Included patients			Excluded patients		
	M	SD	Range	M	SD	Range
Gestational age (weeks)	38.68	1.22	36.26–41.26	38.58	1.82	32.57–41.57
Age at first corrective surgery (months)	3.87	5.52	0–21.86	114	168.25	0–29.89
Duration of hospitalization at first surgery (days)^a^	19.55	19.19	4–127	31.47	43.09	4–305
		*n*			*n*	
Sex, males (%)		42 (64)			119	
Born premature (%)		53 (14)			26 (13)	
Cyanotic CHD (%)^a^		9 (80)	** **	** **	139 (68)	

^a^ Significant difference found between the groups at *p* < .005. CHD, congenital heart disease.

Results for the PSI-4 revealed that the patients' parents' stress levels were not above the clinical threshold and that 33% of them responded defensively to this questionnaire. Considering the potential influence of the defensive responding bias on parent-reported stress (43), we separated the parents who responded defensively to the PSI-4 from those who did not. As expected, we found a significant difference between total stress (*p* < .001, mean raw score of 48 ± 8 vs. 73 ± 15, respectively), PD (*p* < .001, mean raw score of 16 ± 4 vs. 27 ± 7) and DC (*p* < .001, mean raw score of 18 ± 4 vs. 24 ± 7) scores. However, no difference was found when comparing the PCDI scores (*p* = .984, mean raw score of 20 ± 6–21 for both groups). These results suggest that a variety of patterns could potentially emerge in our analyses, when considering the parents’ defensive responding. Thus, Defensive Responding was included as a controlled variable in all regression models. We also compared the PSI-4-SF scores of the patients’ parents who completed the questionnaire before their child underwent surgery to that of parents who completed it after the surgical intervention. No difference was found between the two groups in regard to the Total Stress score (*p* = .952, mean raw score of 64 ± 18 vs. 65 ± 18) nor any of the three subscale scores (PD, *p* = .474, mean score of 25 ± 7 vs. 23 ± 8; PCDI, *p* = .081, mean score of 25 ± 24 vs. 19 ± 8; DC, *p* = .558, mean score of 23 ± 7 vs. 22 ± 7). No difference was found when comparing PSI Total Stress scores of parents who received an antenatal diagnosis of CHD to that of parents who received the diagnosis after birth (*p* = .969, mean score of 65 ± 16–18 for both groups).

### 3.2. Correlations and multiple linear regressions

Bivariate Pearson correlation analyses were performed between the variables of interest (see Table 2). When predicting anxiety symptoms at 24 months (CBCL) while controlling for the patient's sex, socioeconomic status and duration of hospitalization at first corrective cardiac surgery, we found that the regression model built with the PSI-4 DC (Difficult Child) score explained 17% (*R*^2^ = .166; *p* = .048) of the CBCL Anxiety Problems subscale score. In fact, a closer look at the contribution of each variable included in the model shows that the DC subscale score accounts for 36% (*ß* = .358; *p* = .004) of the variance in parent-reported anxiety symptoms at 24 months, independently of controlled variables. No significant predictive value of the PSI-4 PD, PCDI and total stress scores on CBCL Anxiety Problems at 24 months was found. No significant predictive value of PSI-4 scores on the HIBOU scores at 24 months was found either with any of the four regression models (PSI-4 total stress, PD, PCDI and DC). See Table 3 for the detailed results of the linear regression analyses.

**Table 2:** Pearson bivariate correlation matrix between PSI scores at 4 months and CBCL anxiety scale and hibou scores at 24 months.

4- and 24-months scores		24-months scores			
		CBCL Anxiety	HIBOU
PSI-4-SF Total Stress	*r*	0.242	*p* = .050	0.039	*p* = .758
PSI-4-SF PD	*r*	0.160	*p* = .199	0.002	*p* = .990
PSI-4-SF PCDI	*r*	−0.420	*p* = .741	−0.125	*p* = .316
PSI-4-SF DC	*r*	**0** **.** **354**	***p* = .004**	0.161	*p* = .196
Def Resp	*r*	0.188	*p* = .130	−0.006	*p* = .962
HIBOU	*r*	**0**.**274**	***p* = .026**	** **	** **

HIBOU, *Échelle de dépistage des troubles du sommeil pédiatriques*, Pediatric Sleep Disorders Screening Scale; CBCL, Child Behavior Checklist 1.5-5 years old; PSI-4-SF, Parenting Stress Index, 4th Ed., Short Form; PD, Parental Distress; PCDI, Parent-Child Dysfunctional Interaction; DC, Difficult Child; Def Resp, Defensive Responding.

Bold values represent significant results at *p* < .05.

**Table 3:** Results of multiple linear regression analyses between 4- and 24-months scores.

Regression models (PSI-4-SF at 4 months)		24-months CBCL Anxiety score	Regression models (PSI-4-SF at 4 months)		24-months HIBOU score
1. Sex	*β*	−0.050	5. Sex	*β*	−0.124
Socioeconomic status	*β*	−0.083	Socioeconomic status	*β*	−0.025
Duration of hospitalization	*β*	0.183	Duration of hospitalization	*β*	0,168
PSI-4-SF Total Stress	*β*	0.248	PSI-4-SF Total Stress	*β*	0.038
Defensive Responding	*β*	0.041	Defensive Responding	*β*	−0.073
	*R* ^2^	0.096		*R* ^2^	0.048
2. Sex	*β*	−0.050	6. Sex	*β*	−0.124
Socioeconomic status	*β*	−0.083	Socioeconomic status	*β*	−0.025
Duration of hospitalization	*β*	0.183	Duration of hospitalization	*β*	0,168
PSI-4-SF PD	*β*	0.155	PSI-4-SF PD	*β*	−0.027
Defensive Responding	*β*	0.156	Defensive Responding	*β*	−0.001
	*R* ^2^	0.074		*R* ^2^	0.045
3. Sex	*β*	−0.050	7. Sex	*β*	−0.124
Socioeconomic status	*β*	−0.083	Socioeconomic status	*β*	−0.025
Duration of hospitalization	*β*	0.183	Duration of hospitalization	*β*	0,168
PSI-4-SF PCDI	*β*	−0.034	PSI-4-SF PCDI	*β*	−0.121
Defensive Responding	*β*	0.179	Defensive Responding	*β*	−0.018
	*R* ^2^	0.074		*R* ^2^	0.059
4. Sex	*β*	−0.050	8. Sex	*β*	−0.124
Socioeconomic status	*β*	−0.083	Socioeconomic status	*β*	−0.025
Duration of hospitalization	*β*	0.183	Duration of hospitalization	*β*	0,168
PSI-4-SF DC	*β*	**0** **.** **358** **	PSI-4-SF DC	*β*	0.165
Defensive Responding	*β*	0.038	Defensive Responding	*β*	−0.103
	*R* ^2^	**0**.166*		*R* ^2^	0.079

CBCL, Child Behavior Checklist 1.5-5 years old; HIBOU, *Échelle de dépistage des troubles du sommeil pédiatriques*, Pediatric Sleep Disorders Screening Scale; PSI-4-SF, Parenting Stress Index, 4th Ed., Short Form; PD, Parental Distress; PCDI, Parent-Child Dysfunctional Interaction; DC, Difficult Child.

Bold values represent significant results at two different thresholds (indicated at the bottom of the table).

* *p* < 0.05.

** *p* < 0.005.

## 4. Discussion

This longitudinal retrospective study aimed to investigate the predictive value of parenting stress at 4 months on anxiety outcomes and quality of sleep at 24 months in toddlers with CHD. Our results partly align with the hypothesis that was put forward. First, parenting stress scores at 4 months were significantly associated with the child's anxiety symptoms at 24 months. In fact, parenting stress related to the parent's perception of their child's behavioral problems and poor psychosocial adjustment (DC subscale of the PSI-4) acted as a significant predictor of parent-reported anxiety in their toddler at 24 months. Even when controlling for the child's sex, the family's socioeconomic status, the duration of hospitalization at first corrective cardiac surgery and the Defensive Responding bias, the regression model remained significant. This result suggests that parenting stress is an independent predictor of ulterior anxiety in the child, above and beyond sociodemographic and medical factors known to impact the development of children with CHD (44, 45). Karimzadeh et al. (46) have explained that increased levels of parenting stress could have such an impact through a lack of emotional availability or a tendency to assume that the situation is about to spiral out of control when facing any changes or difficulties with the child, even when minor. The PSI-4 DC subscale notably evaluates the parents' perception of their child's ability to self-regulate and cope with adversity (28). Significant maladaptation and increased levels of anxiety in parents could therefore lead to similar ulterior reactions in children through a number of parental psychosocial factors.

Among the four scores obtained with the PSI-4 (Total stress score, PD, PCDI, and DC subscale scores), only the DC score was significantly correlated with the CBCL Anxiety Problems subscale score at 24 months. Since the total stress score represents the sum of the three dimensions of the PSI-4 scale, it is less specific than the three subscale scores considered individually. Perhaps parents of children with CHD have a generally higher threshold for what they perceive to be stressful, given what they have gone through with their child's medical condition (47), and this could contribute to lowering their self-reports of personal distress (PD subscale). This could alter the predictive value of the total stress score, but not the DC subscale score, which is more oriented towards their worries about the child and proved to significantly predict anxiety at 24 months. To better explain this disparity between the predictive value of the parenting stress subscale scores, we considered the proportion of parents who tended to respond defensively to the questionnaire, which was particularly high in our sample (33%). Social desirability and denial of their child's precarious condition are two of many hypotheses that could explain the parents' defensiveness. Moreover, defensiveness seems to be more frequent in clinical settings, and especially so when the child's situation is particularly difficult and precarious, which is the case for our cardiac neurodevelopmental follow-up clinic's patients (43). Hence, considering the many medical-related factors that could contribute to the parents' anxiety and their impact on the tendency to answer defensively, it is possible that parenting stress levels might have been underestimated in our study. We can also hypothesize that the defensive attitude towards parent-reported questionnaires is more specific to parent-oriented variables, such as the PD and PCDI subscales, which in turn suggests that child-oriented subscales, such as the DC subscale, might be more accurate in describing actual parenting stress levels. This underlines the importance of additional measures to corroborate self-report findings and compensate for defensive responding, since few standardized tools are designed to detect it (48).

Finally, parenting stress scores at 4 months were not associated with the child's perceived quality of sleep at 24 months. This could be due to the broad nature of the HIBOU screening tool, as explained by Martin et al. (49) in their meta-analysis on the interplay between parenting stress and sleep difficulties in children with autism spectrum disorder and attention deficit/hyperactivity disorder. The HIBOU offers a composite score that combines many types of sleep problems which are not individually explored in detail, making it impossible to assess a specific deficit independently, nor its interplay with our other variables of interest. Although objective data obtained through polysomnography is preferable when objectively assessing sleep quality (49), primarily subjective measures such as parent-reported sleep questionnaires act as objective measures in clinical contexts. This is particularly true when the questionnaires have been adapted to clinical populations (50, 51), as for the HIBOU. Nevertheless, we found a significant correlation between the HIBOU score and CBCL Anxiety Problems subscale score at 24 months, demonstrating a relationship between the two variables, possibly through common predicting factors, which is consistent with Chorney et al.'s (52) literature review. Indeed, the authors recommend assessing symptoms of disturbed sleep when measuring anxiety symptoms in children. Though we use the HIBOU to assess sleep at our cardiac neurodevelopmental follow-up clinic, more detailed and specific parent reports, as well as objective sleep data, should be considered.

### 4.1. Clinical implications

Although the parents do not necessarily report clinically significant parenting stress levels, probably due to the high rate of significant Defensive Responding Indices, our results show that, when administered early, parenting stress scores could have a predictive value of the child's ulterior anxiety during toddlerhood (even when controlling for the tendency to answer defensively). The PSI scores could be used for early identification of parents and children potentially at risk of more important behavioral and psychoaffective challenges, which are difficult to assess with standardized tools at this young age (53). At our cardiac neurodevelopmental follow-up clinic, systematic interdisciplinary follow-up and psychosocial care (including systematic assessments of parenting stress) are offered when children with CHD reach the age of 4 months. Yet, an important proportion of families receive the diagnosis during pregnancy and carry this burden for several weeks before the child is born. Since prenatal stress usually evolves into parenting stress after birth (54), its adverse effects tend to consolidate.

We also found that parenting stress scores at 4 months and the child's perceived quality of sleep at 24 months were both significantly associated with anxiety problems at 24 months. Since sleep difficulties are common in congenital heart disease (55), we must work on informing the parents on the relationship between sleep and anxiety, and promoting healthy sleep habits once the child leaves the hospital. Because stress is known to be significantly high in parents of children with CHD, and influenced by sleep difficulties in those children (7–15), it must also be considered as one of the important targets of the psychosocial care and interventions offered to families (22).

### 4.2. Methodological considerations

The current study has limitations that must be considered. First, significant differences were found between participants and non-participants regarding two medically-related variables: a smaller proportion of perinatal diagnosis and a shorter average duration of hospitalization at first corrective cardiac surgery were both found in our sample. Since an antenatal diagnosis is associated with more complex forms of CHD (56), we hypothesize that those parents might be even more diligent in completing questionnaires (especially at 4 months) due to the anxiety caused by the increased risk of post-surgery complications. Parents of children with less severe forms of CHD could therefore have been excluded from the study right from the start, making our results less generalizable to the population (only 30% of the eligible patients' parents had completed all questionnaires). On an exploratory basis, we incorporated the time of diagnosis ante- vs. perinatal) in our linear regression analyses, by replacing the duration of hospitalization at first corrective cardiac surgery (controlled variable for all models). No significant differences were found, meaning that the time of diagnosis does not seem to affect the predictive value of parenting stress on the child's ulterior anxiety. The role of other clinical factors related to CHD is also extremely relevant to investigate and should be considered in further research with an adequate statistical power, allowing to include these variables.

Second, all the questionnaires that we used are standardized except the HIBOU, which provides a measure of the parent's perception of the child's quality of sleep. Nonetheless, it is widely used in both clinical and research contexts throughout the province of Quebec. Its scoring procedure provides a relevant clinical cut-off and allows for early identification of potential sleep disturbances. We put forward the interesting alternative of an at-home sleep monitoring device that would provide objective data on specific parameters of the child's sleep (i.e., oxygen saturation, heart rate, etc.). Using a device that targets cardiorespiratory parameters, Vézina et al. (57) obtained clinically acceptable data in 91% of their 562 healthy 1-year-olds, proving the efficacy of such a device with infants. In a future study, the device could be provided to patients' families at specific times during the systematic follow-up sequence for recordings of one night. Objective and standardized data on the child's sleep could then be compared to the results of parent-reported questionnaires. Monitoring the parents' sleep would also be relevant in further research, given its interplay with the child's quality of sleep and its influence on anxiety symptoms.

## 5. Conclusions

The present study shows that parenting stress can act as a predictor of anxiety symptoms in toddlers with CHD. Our results revealed that the parents’ perception of their child's poor behavioral and psychosocial adjustment at 4 months of age contributed significantly to the prediction of ulterior anxiety at 24 months. Accurate measures of the patients' parents' stress levels could therefore allow for early identification of distressed families. However, the defensive responding bias must be considered, as it might lead to an underestimation of the parenting stress levels during the first months of life. Normal or lower-than-normal stress levels therefore do not necessarily indicate a healthy adaptation to life with the disease, and measures that are more oriented towards the parents’ worries about the child, instead of toward themselves, might be more appropriate. The results of this study also bring into consideration the necessity for very early intervention. For families who receive an antenatal diagnosis of CHD, antenatal psychosocial interventions, such as stress management workshops, psychotherapy or psychoeducation follow-up sessions, should be considered in order to optimize developmental outcomes.

## Data Availability

The raw data supporting the conclusions of this article will be made available by the authors, without undue reservation.
