# Supplementary material for: The role of parenting stress in anxiety and sleep outcomes in toddlers with congenital heart disease
Source: Front Pediatr. 2023 Jan 6;10:1055526. doi: 10.3389/fped.2022.1055526 (PMC9853386; doi:10.3389/fped.2022.1055526)
Supplement: Supplementary file 1 [file Table1.docx]

| Variables  ***Supplementary Material***  **Table 1.** Descriptive statistics and sociodemographic characteristics of participants | n (%)^a^ | M (SD) | Range |
| --- | --- | --- | --- |
| *Sociodemographic factors* |  |  |  |
| Sex, males (% of sample) | 42 (63.64) |  |  |
| Age when research was conducted (months) | 66 | 43.16 (7.07) | 28-58 |
| Socioeconomic status score^b^, mother | 66 | 60.22 (22.46) | 12-100 |
| Socioeconomic status score, father | 66 | 59.23 (24.49) | 10-96 |
| Socioeconomic status score, average | 66 | 59.72 (20.23) | 12-92 |
| *Medical factors* |  |  |  |
| Premature birth (% of sample) | 9 (13.64) |  |  |
| Gestational age (weeks) | 66 | 38.68 (1.22) | 36.26-41.26 |
| Time of diagnosis, antenatal (%) | 56 (84.85) |  |  |
| Cyanotic cardiac anomaly (%) | 53 (80.30) |  |  |
| Age at first corrective surgery (months) | 66 | 3.87 (5.52) | 0-21.86 |
| Severe cardiac anomaly | 63 (95) |  |  |
| Moderate cardiac anomaly | 3 (5) |  |  |
| Surgical risk category^c^ at first corrective surgery |  |  |  |
| R1 | 0 |  |  |
| R2 | 39 (59) |  |  |
| R3 | 19 (27) |  |  |
| R4 | 4 (6) |  |  |
| R5 | 0 |  |  |
| R6 | 3 (5) |  |  |
| Duration of hospitalization (days) | 66 | 19.55 (19.19) | 4-127 |
| Duration of NICU/PICU stay (days) | 65 | 8.42 (9.11) | 2-66 |
| No. of corrective surgeries before 24 months = 1 (%) | 52 (78.79) |  |  |
| No. of corrective surgeries before 24 months = 2 (%) | 10 (15.15) |  |  |
| No. of corrective surgeries before 24 months = 3 (%) | 3 (4.55) |  |  |
| *Parenting stress* |  |  |  |
| PSI-4-SF Total Stress raw score (max 180) | 66 | 64.83 (17.83) | 39-102 |
| PSI-4-SF PD raw score (max 60) | 66 | 23.24 (7.72) | 12-44 |
| PSI-4-SF PCDI raw score (max 60) | 66 | 18.76 (5.92) | 12-36 |
| PSI-4-SF DC raw score (max 60) | 66 | 22.47 (6.87) | 13-37 |
| Above clinical threshold of 110 (%) | 0 (0) |  |  |
| Defensive Responding (%) | 22 (33.33) |  |  |
| Completed by mother (%) | 46 (74.19) |  |  |
| Completion age (months) | 66 | 5.05 (2.88) | 2.20-17.65 |
| Completion before child's first corrective surgery (%) | 17 (25.76) |  |  |
| Interval between completion and first corrective surgery (days) | 66 | 134.23 (112.42) | 2-537 |
| *Sleep* |  |  |  |
| HIBOU raw score (max 27) | 66 | 5 (3.65) | 0-13 |
| Above clinical threshold of 10 (%) | 9 (13.64) |  |  |
| Completed by mother (%) | 60 (90.90) |  |  |
| Completion age (months) | 66 | 24.24 (0.93) | 23.08-29.03 |
| *Anxiety symptoms* |  |  |  |
| CBCL 1.5-5 years old Anxiety raw score (max 26) | 66 | 2 (2) | 0-10 |
| Above clinical threshold of 7 (%) | 2 (3.03) |  |  |
| Completed by mother (%) | 57 (86.36) |  |  |
| Completion age (months) | 66 | 24.16 (0.92) | 23.01-29.03 |
| ^a^n refers to the number of patients for which specific nominal/ordinal characteristics were applicable (with %) or the number of patients for which the interval data was available (no %) ^b^Socioeconomic status scores calculated using Boyd's Socioeconomic Scale for Canada (2008). Severity of cardiac anomaly was determined using Hoffman and Kaplan’s definitions (2002). Surgical risk category was determined using Jenkins’ classification model (RACHS; 2002). Abbreviations: NICU/PICU, Neonatal/Pediatric Intensive Care Unit; HIBOU, *Échelle de dépistage des troubles du sommeil pédiatriques*, Pediatric Sleep Disorders Screening Scale; CBCL, Child Behavior Checklist 1.5-5 years old; PSI-4-SF, Parenting Stress Index, 4th Ed., Short Form; PD, Parental Distress; PCDI, Parent-Child Dysfunctional Interaction; DC, Difficult Child; M, mean; SD, standard deviation. | | | |
